# Hyaluronic Acid Modified Nanostructured Lipid Carrier for Targeting Delivery of Kaempferol to NSCLC: Preparation, Optimization, Characterization, and Performance Evaluation In Vitro

**DOI:** 10.3390/molecules27144553

**Published:** 2022-07-17

**Authors:** Yufei Ma, Jinli Liu, Xinyu Cui, Jiafu Hou, Fengbo Yu, Jinghua Wang, Xiaoxue Wang, Cong Chen, Lei Tong

**Affiliations:** 1 Department of Pharmacy, Mudanjiang Medical University, Mudanjiang 157000, China; mayufei693@126.com (Y.M.); houjiafu@mdjmu.edu.cn (J.H.); yfb526@126.com (F.Y.); wjh6466@163.com (J.W.); 18745506531@163.com (X.W.); c1449320686@163.com (C.C.); 2 Department of Basic Medicine, Mudanjiang Medical University, Mudanjiang 157000, China; liujinli1986123@163.com; 3 Department of Public Health, Mudanjiang Medical University, Mudanjiang 157000, China; xinyucui@outlook.com

**Keywords:** nanostructured lipid carrier, kaempferol, non-small cell lung cancer

## Abstract

Lung cancer seriously threatens the health of human beings, with non-small cell lung cancer (NSCLC) accounting for 80%. Nowadays, the potential position of nano-delivery in treating cancer has been the subject of continuous research. The present research aimed to prepare two molecular weight hyaluronic acid (HA)-modified kaempferol (KA)-loaded nanostructured lipid carriers (HA-KA-NLCs) by the method of melting ultrasonic and electrostatic adsorption, and to assess the antitumor effect of the preparations on A549 cells. The characterization and safety evaluation of the preparations illustrated that they are acceptable for drug delivery for cancer. Subsequently, differential scanning calorimetry (DSC) curve and transmission electron microscopy (TEM) images indicated that the drug was adequately incorporated in the carrier, and the particle appeared as a sphere. Moreover, HA-KA-NLC showed predominant in vitro antitumor effects, inhibiting proliferation, migration, and invasion, promoting apoptosis and increasing cellular uptake of A549 cells. Otherwise, the Western blot assay revealed that preparations could activate epithelial-mesenchymal transition (EMT)-related signaling pathways and modulate the expression of E-cadherin, N-cadherin, and Vimentin in A549 cells. Our present findings demonstrated that HA-KA-NLC could be considered as a secure and effective carrier for targeted tumor delivery and may have potential application prospects in future clinic therapy of NSCLC.

## 1. Introduction

Globally, lung cancer ranks first in malignant tumors, with the highest incidence and mortality, and it easily metastasizes to the bones [1], resulting in additional tumors [2]. Non-small cell lung cancer (NSCLC) makes up about 80% of lung cancer cases and is the most widespread and refractory pathological type [3]. NSCLC patients always have a poor prognosis and a low five-year survival rate [4]. Clinically, chemotherapy [5], radiation [6], and surgery are the common treatments for NSCLC, but they ordinarily have systemic side effects that limit the therapeutic efficacy.

Natural components have been gradually introduced for the treatment of tumors in clinic, not only improving the life quality of cancerous patients but also reducing harmful and toxic effects [7,8]. Kaempferol (KA) is a polyphenolic flavonoid that can be found in multifarious natural substances, such as grapefruit, gingko, tea, propolis, and so on, and displays various medicinal and biological properties [9]. Numerous studies have suggested that KA has a wealth of health benefits, including antibacterial [10], anti-asthmatic [11], anti-inflammatory [12], stroke prevention [13], antioxidant [14], hypoglycemic [15], and atherosclerosis preventive [16]. According to the previous studies, KA is an effective antitumor drug in suppressing migration and invasion, modulating the genome, and promoting apoptosis of NSCLC by regulating various signal transduction cellular pathways [17,18,19,20]. Nevertheless, its low aqueous solubility and poor oral bioavailability severely limit the clinical application, which needs to be addressed through innovative pharmaceutical techniques [21,22].

Recently, nanocarriers have contributed to overcome biopharmaceutical drawbacks of drugs by modifying drug release and distribution, including nanoemulsions [23], microemulsions [24], liposomes [25], and solid lipid nanoparticles [26]. As the third generation of lipid nanoparticles, the nanostructured lipid carrier (NLC) is recognized as a high-efficiency carrier, since it achieves the controlled or sustained release of the insoluble substance [27,28]. Meanwhile, NLC has higher encapsulation efficiency (EE) and drug loading (DL) than other preparations due to the irregular crystal structure that uses a mixture of solid lipid and liquid lipid [29]. Hence, the drug incorporated into NLC formulations has the extreme potential to ameliorate drug defects and alter the pharmacokinetics, which could enhance therapeutic efficacy [30].

In order to further improve the efficacious treatment of NSCLC, the targeting factor is commonly used for modification. Hyaluronic acid (HA) is a negatively charged linear polysaccharide ubiquitously existing in the extracellular matrix [31]. HA has been used in drug delivery, disease diagnosis, and biomedical imaging as a potential material [32,33]. CD44, an adhesion protein overexpressed in NSCLC cells, is correlated with tumorigenesis, proliferation, invasion, and malignant features [34,35]. CD44 is known as a special receptor for HA; thus, the selective interaction can be used for targeting cancer and leading the drug to intracellular regions [36,37]. As a result, HA was chosen as the targeted factor for treatment based on the pathological features of NSCLC cells, hence improving therapeutic effectiveness and lowering systemic toxicity. Since different molecular weights of HA possess diverse biological activities [38], we considered whether different molecular weights of HA have varied targeting effects. With this in mind, we modified HA with two distinct molecular weights and compared the in vitro antitumor effects in this investigation.

**Figure 1:**
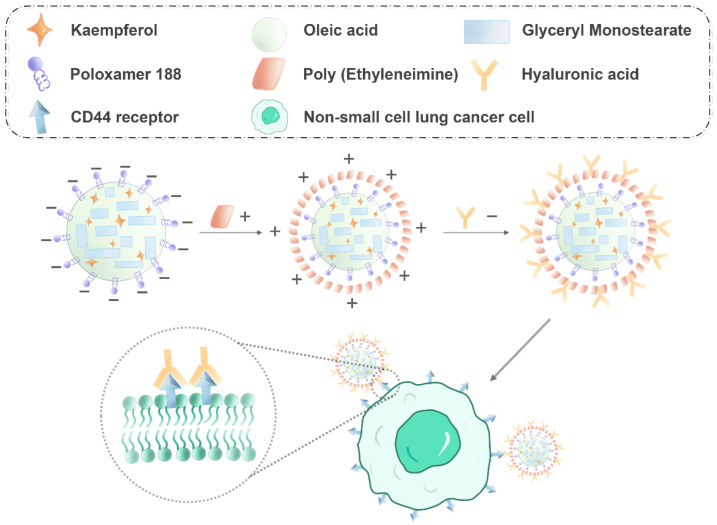
Schematic diagram of HA-KA-NLC for NSCLC cell treatment (the minus and plus signs represent negative and positive charges, respectively).

The current study aimed to develop KA-NLC modified by HA for two molecular weights to obtain HA_20_-KA-NLC (HA, M.W. 200–400 kDa) and HA_130_-KA-NLC (HA, M.W. 1300–1600 kDa) for targeting NSCLC (Figure 1). Subsequently, their in vitro antitumor effects against A549 cells were evaluated.

## 2. Results

### 2.1. Optimization Analysis Using the BBD Method

**Figure 2:**
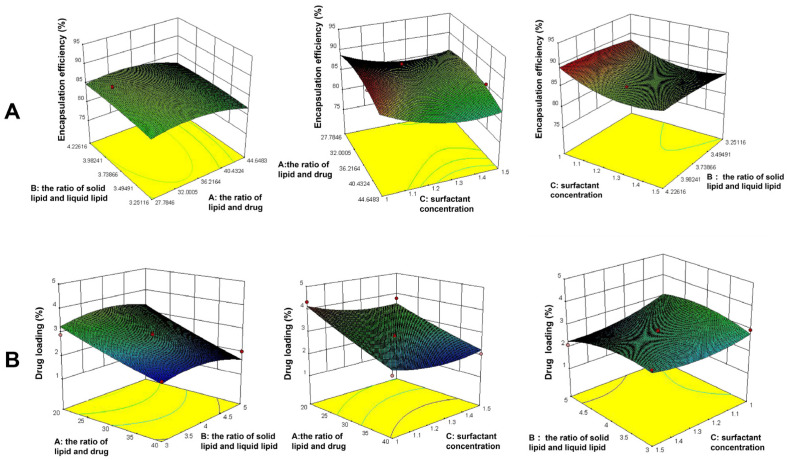
The influence of independent constraints on (**A**) encapsulation efficiency and (**B**) drug loading in 3D response surface plots.

The effect of critical factors was observed on dependent variables like EE% and DL%; a total of 17 experiments are listed in Table 1. The 3D response surface plots are shown in Figure 2. The following regression equations express the mathematical relationship between three independent variables and two dependent variables.

The model equation of EE% is as follows: Y_1_ = 85.49 + 1.83 A + 0.099 B − 1.67 C − 0.28 AB − 0.57 AC − 0.050 BC − 2.54 A^2^ − 1.71 B^2^ + 2.30 C^2^. (R^2^ = 0.9478).

The model equation of DL% is as follows: Y_2_ = 2.79 − 0.74 A − 0.054 B − 0.21 C − 0.085 AB + 0.095 AC − 0.20 BC + 0.055 A^2^ − 0.31 B^2^ + 0.25 C^2^. (R^2^ = 0.9096).

The statistical significance of the regression model was checked by p-value. The results showed that A, C, A^2^, B^2^, and C^2^ had a significant effect on EE% (*p* < 0.05), while A had a significant effect on the DL% (*p* < 0.05). A synergistic impact is shown by a positive sign, whereas an antagonistic effect is indicated by a negative sign [39]. The results derived from the experiment runs, the optimized formulation for KA-loaded NLC was prepared as follows: the ratio of lipid and drug = 34.72:1, the ratio of solid lipid and liquid lipid = 4:1, and surfactant concentration = 1% (*w*/*v*). The particle size, PDI, and Zeta potential of KA-NLC prepared in optimal conditions were 53.2 ± 2.1 nm, 0.274 ± 0.042, and −26.2 ± 1.4 mV, respectively. The particle size of drug delivery is an essential factor, and the particle size (<200 nm) was found to be acceptable for enhancing drug accumulation and increasing systemic circular time because of the enhanced permeability and retention (EPR) effect [40]. The narrow PDI value (0.274) indicated the favorable uniformity of nanoparticle distribution. In addition, the Zeta potential represented the migration velocity of the particles; ±30 mV was instrumental in stabilizing the NLC suspension system.

### 2.2. Characterization of KA-NLC

**Figure 3:**
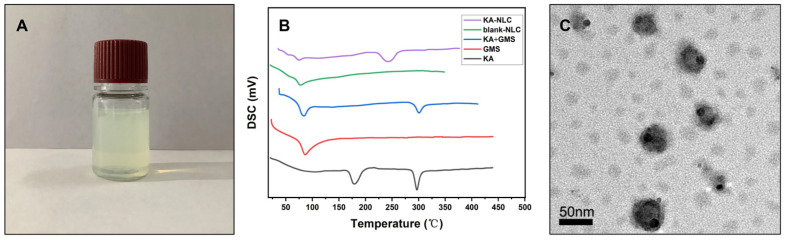
(**A**) Appearance of KA-NLC. (**B**) Thermograms of KA-NLC, blank-NLC, physical mixture of KA and GMS, GMS, and KA. (**C**) TEM images of optimal KA-NLC; the optimal formulation is as follows: the ratio of lipid and drug = 34.72:1, the ratio of solid lipid and liquid lipid = 4:1, and surfactant concentration = 1% (*w*/*v*).

EE% is an essential index of quality evaluation to visually reflect the drug loading capacity of the carrier, which is important for further applications. The EE% of optimized KA-NLC was determined to be 89.22 ± 2.33%, and the DL% was founded to be 2.96 ± 0.56%. According to the DSC thermogram (Figure 3B), the characteristic endothermic peak of GMS and KA were 78 °C and 296 °C, respectively. Meanwhile, the typical peaks of both GMS and KA were observed in the physical mixture of KA and GMS. The DSC curve of KA-NLC showed a peak at an intensity of 236.4 °C, which was slightly less than the intensity of pure KA, indicating the internal crystal structure of KA was devastated. The results confirmed that KA was successfully encapsulated inside the lipid matrix of NLC. Simultaneously, the shape and surface morphology of KA-loaded NLC were evaluated by TEM. The TEM images (Figure 3C) showed a spherical shape and homogeneous dispersion of nanoparticles, which confirmed our method could be successfully used to prepare KA-NLC.

### 2.3. Characterization of HA-KA-NLC

In our study, the negative charge presenting on the surface of KA-NLC was exploited for deposition of the cationic polymer PEI, following a modified layer of the anionic polysaccharide HA. Accordingly, to ensure appropriate absorption of KA-NLC, the amount of PEI and HA required to reverse the charge of nanoparticles in each step was optimized by the change of the Zeta potential procedure.

**Figure 4:**
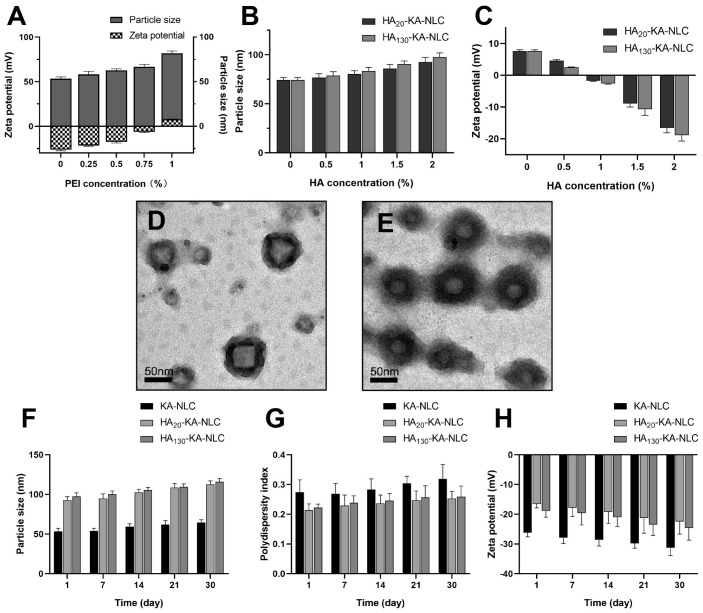
(**A**) Particle size and Zeta potential of preparations with different concentrations of PEI. (**B**) Particle size and (**C**) Zeta potential of HA_20_-KA-NLC and HA_130_-KA-NLC with different concentrations of HA. TEM images of (**D**) HA_20_-KA-NLC and (**E**) HA_130_-KA-NLC. The change of (**F**) particle size, (**G**) PDI, and (**H**) Zeta potential over 30 days of experiments, depending on the storage conditions.

The results are shown in Figure 4A: the increase of PEI led to an inflection point; after the concentration of PEI reached 1%, the Zeta potential was reversed to a positive charge (+7.8 ± 0.7 mV), and the particle size of nanoparticles was 74.3 ± 2.5 nm at this concentration.

When the concentration of HA increased to 2% during the assembly process, the particle size increased to 92.7 ± 4.6 nm and 97.6 ± 4.3 nm in HA_20_-KA-NLC and HA_130_-KA-NLC (Figure 4B), respectively. The increment in particle size after modification by HA was insignificant, as electrostatic interaction between HA and PEI resulted in a stable status with the small particle size. Meanwhile, the surface charge decreased to −16.6 ± 1.5 mV and −18.9 ± 1.8 mV in HA_20_-KA-NLC and HA_130_-KA-NLC (Figure 4C), respectively. Collectively, the noticeable switching of Zeta potential suggested a successive modification and realized a layer by layer structure. Additionally, the TEM images (Figure 4D,E) also overly illustrated that the surface of KA-NLC was abundantly modified by HA.

The stability study results suggested that the change of preparations was inconspicuous when observed with the eye after 30 days. Results are illustrated in the Figure 4F–H; the parameters of KA-NLC, HA_20_-KA-NLC, and HA_130_-KA-NLC fluctuated within a certain range, but within acceptable limits, which showed that the preparations were stable in 30 days.

### 2.4. In Vitro Drug Release Study

**Figure 5:**
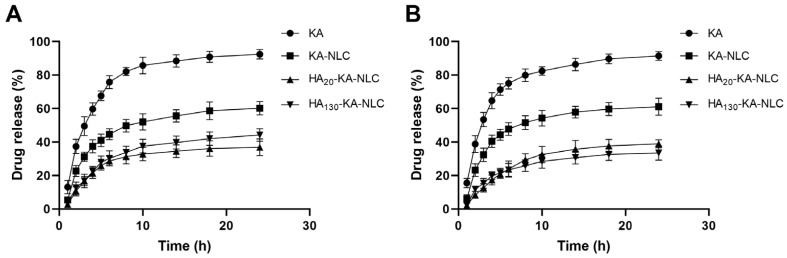
The in vitro drug release of preparations in (**A**) pH 5.5 and (**B**) pH 7.4.

The release pattern of KA, KA-NLC, HA_20_-KA-NLC, and HA_130_-KA-NLC is shown in Figure 5. At the end of 8 h, it was observed that the release of KA was 82.12 ± 2.42% and 79.96 ± 3.74% in pH 5.5 and pH 7.4, respectively, while the release of KA from KA-NLC did not reach 50% at the time both in pH 5.5 and 7.4. The results showed an initial burst release (37.47 ± 3.97% and 40.47 ± 3.68%) of KA-NLC formulation in the first 4 h of the study. It was possible that the unencapsulated drug presented on the surface of NLC. Moreover, the release of KA from HA_20_-KA-NLC and HA_130_-KA-NLC was lower than KA-NLC. This phenomenon might be due to KA being entrapped in the NLC core and the surface being modified by HA, increasing the barriers for the drug release. The sustained release pattern may delay the drug’s exposure in tumor cells, thereby potentially enhancing the curative effect. However, there was no obvious difference in release behavior obtained from HA_20_-KA-NLC and HA_130_-KA-NLC.

### 2.5. Safety Evaluation of Preparations In Vitro

**Figure 6:**
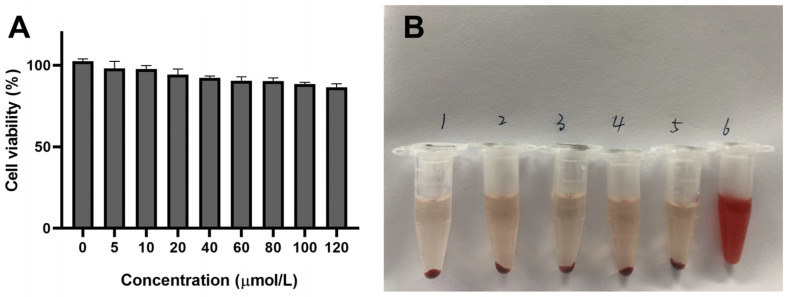
(**A**) In vitro cytotoxicity assay of blank-NLC after 24 h of co-incubation in A549 cells. (**B**) Photo of RBCs mixed with (1) physiological saline, (2) blank-NLC, (3) KA-NLC, (4) HA_20_-KA-NLC, (5) HA_130_-KA-NLC, and (6) ultrapure water for 3 h.

The safety of the delivery carrier is an essential aspect for effective application; thus, the cytotoxicity and hemolysis of preparations were undertaken. The cell viability treated with different concentrations of blank-NLC was above 85% (Figure 6A), which revealed that the NLC we designed is safe and hypotoxic. At the same time, the result of in vitro hemolysis assay (Figure 6B) displayed that the upper layer of the blank-NLC, KA-NLC, HA_20_-KA-NLC, HA_130_-KA-NLC, and the negative control group were approximately colorless with erythrocyte precipitation in the lower layer, while the positive control group was light red. The hemolysis percentage of blank-NLC, KA-NLC, HA_20_-KA-NLC, and HA_130_-KA-NLC groups were 2.44 ± 0.15%, 1.02 ± 0.09%, 2.52 ± 0.28%, and 1.86 ± 0.19%, respectively, indicating no apparent hemolysis effect in the samples, which could be used for subsequent studies.

### 2.6. Evaluation of Cell Viability by CCK-8 Assay

**Figure 7:**
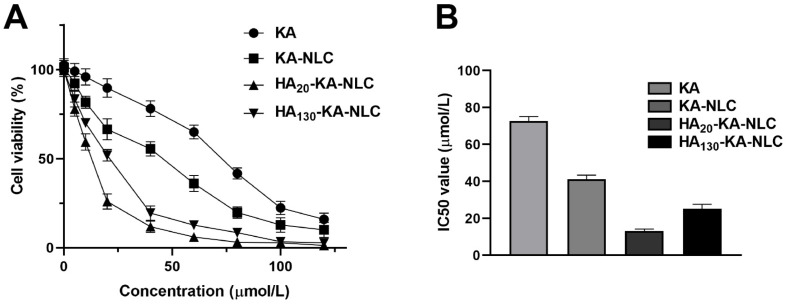
CCK-8 assay was performed in A549 cells with KA, KA-NLC, HA_20_-KA-NLC, and HA_130_-KA-NLC treatments. (**A**) The cell viability treated by different preparations with different concentration in A549 cells. (**B**) The 24 h IC_50_ values in A549 cells treated by different preparations. The data are presented as the mean ± SD (*n* = 3).

The results (Figure 7A) showed that the cell viability has significant concentration dependence, in the range of 0.01–100 µmol/L in A549 cells. The cell viability of KA showed no or very little decline at low concentration due to its poor water solubility. Meanwhile, KA-NLC, HA_20_-KA-NLC, and HA_130_-KA-NLC showed remarkable inhibiting viability effects toward A549 cells at all concentrations compared with the control group. Moreover, the IC_50_ value (Figure 7B) of KA, KA-NLC, HA_20_-KA-NLC, and HA_130_-KA-NLC with treatment for 24 h in A549 cells was 72.8, 41.23, 13.17, and 24.54 µmol/L, respectively. The decline of the IC_50_ value of KA-NLC illustrated an evident enhancement in inhibiting cell proliferation. The IC_50_ value significantly decreased in HA_20_-KA-NLC and HA_130_-KA-NLC compared with KA-NLC, which indicated that modification by HA decreased the cell viability. The enhanced cell proliferation inhibition of HA_20_-KA-NLC and HA_130_-KA-NLC may be associated with the special recognition of HA to the CD44 receptor, which increased the intracellular drug accumulation. Nevertheless, it is apparent that HA_20_-KA-NLC greatly decreased the proliferating capacity.

### 2.7. EdU Staining Assay for the Evaluation of Cell Proliferation

**Figure 8:**
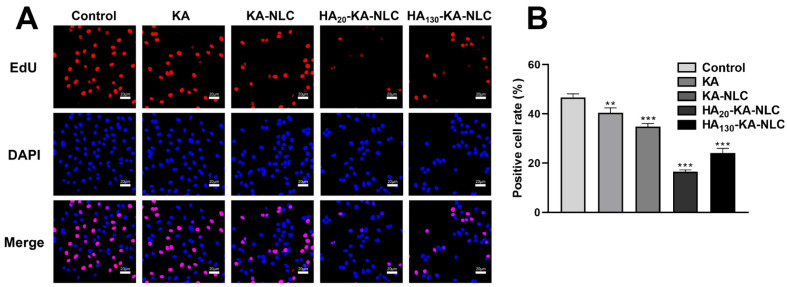
EdU proliferation assay performed in A549 cells without treatment or with KA, KA-NLC, HA_20_-KA-NLC, and HA_130_-KA-NLC treatments. (**A**) Representative images of EdU staining assays. (**B**) Positive cell rate after different treatments. The data are presented as the mean ± SD (*n* = 3). ** *p* < 0.01, *** *p* < 0.001.

Following CCK-8 assay, we performed an EdU assay to further observe the inhibitory ability in cell proliferation of different preparations. In the proliferation images (Figure 8), red is the nucleus in the proliferative state, and blue is the nucleus of all cells. The positive cell rate of control, KA, KA-NLC, HA_20_-KA-NLC, and HA_130_-KA-NLC were 46.70%, 40.68%, 34.82%, 16.20%, and 23.75% in A549 cells, respectively. The results illustrate that the treatment of KA-NLC decreased the positive cell rate compared with KA, and the modification of HA significantly decreased the positive cell rate. Taken together, the above results show that the preparations have the significant ability of inhibiting cell proliferation, and HA_20_-KA-NLC has the best antitumor activity.

### 2.8. Clone Formation Assay for the Evaluation of Cell Proliferation

**Figure 9:**
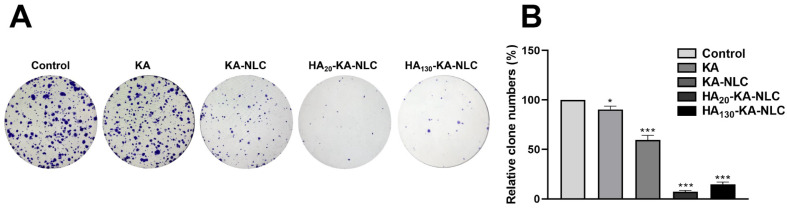
Clone formation assay conducted on A549 cells without treatment or with KA, KA-NLC, HA_20_-KA-NLC, and HA_130_-KA-NLC treatments. (**A**) Representative images of clone formation assays. (**B**) Relative clone numbers after different treatments. The data are presented as the mean ± SD (*n* = 3). * *p* < 0.05, *** *p* < 0.001.

For evaluating the effect of the different preparations on the activity of the cells, the clone formation assay was applied. Consistent with the cell viability assay and EdU assay, the result of the clone formation assay (Figure 9) indicated that the number of cell clones treated by KA-NLC was reduced compared with that by KA. However, the formation of clones further declined due to the HA modified on KA-NLC, but the inhibitory effect of clone formation of HA_20_-KA-NLC was slightly stronger than HA_130_-KA-NLC, which was accordant with the results of the CCK-8 and EdU assay.

### 2.9. Evaluation of Cell Migration by Wound Healing Assay

**Figure 10:**
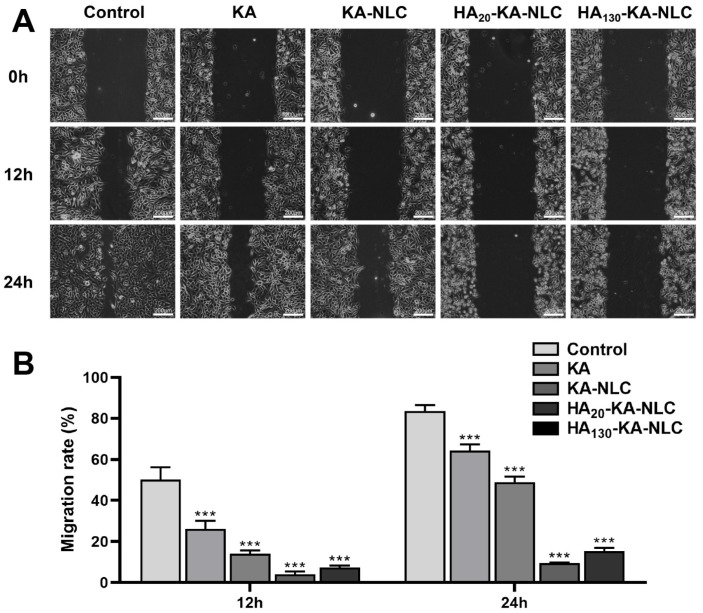
Migration assay conducted on A549 cells without treatment or with KA, KA-NLC, HA_20_-KA-NLC, and HA_130_-KA-NLC treatments. (**A**) Representative images of wound healing assays. (**B**) Migration rate of cells after different treatments. The data are presented as the mean ± SD (*n* = 3). *** *p* < 0.001.

The migratory ability of cells was reflected by the wound area with different preparation treatments. As shown in Figure 10, the cell migration rate of the control group was 83.66% in A549 cells at 24 h, showing the wound had nearly healed. Nonetheless, the KA-NLC group had a lower migration rate compared with KA group at 12 h and 24 h, indicating that the nanosystem effectively improved the problem of poor water solubility of KA. Furthermore, the migration rate in HA_20_-KA-NLC and HA_130_-KA-NLC groups were lower than in the KA-NLC group both at 12 h and 24 h, which clearly confirmed the role of HA in targeting the A549 cells. However, the inhibitory effect of cell migration in the HA_20_-KA-NLC group was stronger than that in the HA_130_-KA-NLC group in A549 cells.

### 2.10. Evaluation of Cell Migration and Invasion by Transwell Assay

**Figure 11:**
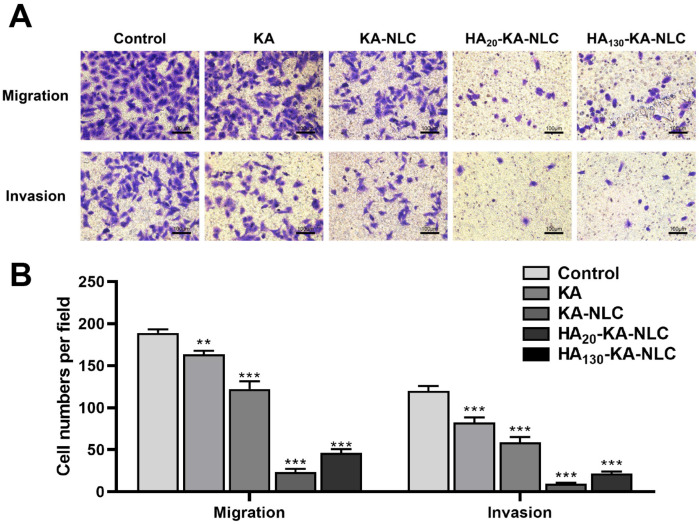
Transwell assay conducted on A549 cells without treatment or with KA, KA-NLC, HA_20_-KA-NLC, and HA_130_-KA-NLC treatments. (**A**) Representative images of migration and invasion assay. (**B**) Number of migratory or invasive cells per field after different treatments. The data are presented as the mean ± SD (*n* = 3). ** *p* < 0.01, *** *p* < 0.001.

NSCLC cells generally have strong migration and invasion ability, which is an essential indicator of tumor metastasis in vivo; thus, we used 24 h Transwell assay to evaluate the function of the drug delivery carrier in inhibiting cell migration and invasion. From Figure 11, it can be seen that the migrated cell numbers in the KA group declined compared with the control group, and the migrated cell numbers further declined in KA-NLC group. The capability of cell migration obviously declined in the HA_20_-KA-NLC and HA_130_-KA-NLC groups, and the difference was statistically significant compared with KA-NLC. Moreover, the results of the invasion assay were similar to those of the migration assay. This may have relevance to preparations that could inhibit the spread of cells, enhance the intercellular adhesion ability, and prevent the movement of tumor cells by inhibiting the EMT process of NSCLC cells. Nonetheless, the targeting delivery effect of HA_20_-KA-NLC was more predominant than that of HA_130_-KA-NLC in A549 cells both in migration and invasion assays.

### 2.11. Analysis of Apoptosis Activity by Hoechst Staining Assay

**Figure 12:**
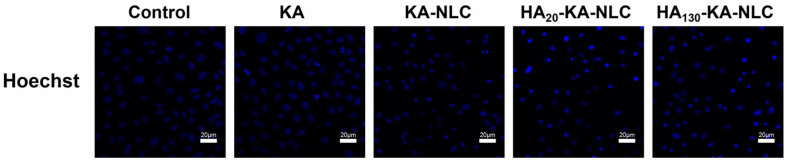
Cellular apoptosis assay conducted on A549 cells without treatment or with KA, KA-NLC, HA_20_-KA-NLC, and HA_130_-KA-NLC treatments.

Hoechst staining assay was conducted to judge the apoptosis effects of the KA-loaded formulations. The images are shown in Figure 12. There was no substantial alteration in KA and control groups; the nuclei visualized feebly and homogenously with blue color. However, the phenomenon of little nuclear fragmentation and shrinkage in the nucleus appeared in the KA-NLC group. In contrast, the number of apoptosis cells conspicuously increased in HA_20_-KA-NLC and HA_130_-KA-NLC groups, and the HA_20_-KA-NLC group increased the proportion of cells that had some typical apoptosis features, with obvious nuclei pyknosis or karyolysis [41]. This phenomenon indicated that the modification of HA enhanced the accumulation of KA in cells and induced the apoptosis of cells, which could be the reason for the high selectivity of HA toward NSCLC cells; however, the mechanism needs to be further explored. To summarize, HA_20_-KA-NLC showed the best targeted capability among all preparations, as evidenced by the above assays.

### 2.12. Cellular Uptake of Different Preparations

To assess the efficacy of the preparations modified by HA in NSCLC cells that CD44 overexpressed, we performed the cellular uptake experiments. The cellular uptake assay can circumstantially prove the advantages of the preparations, including the effect of drug delivery in improving drug defects and enhancing the targeted efficiency. The drug concentration used in this experiment was 40 µmol/L, since the survival rates of cells treated with CUR, CUR-NLC, HA_20_-CUR-NLC, and HA_130_-CUR-NLC were greater than 80% at this concentration.

**Figure 13:**
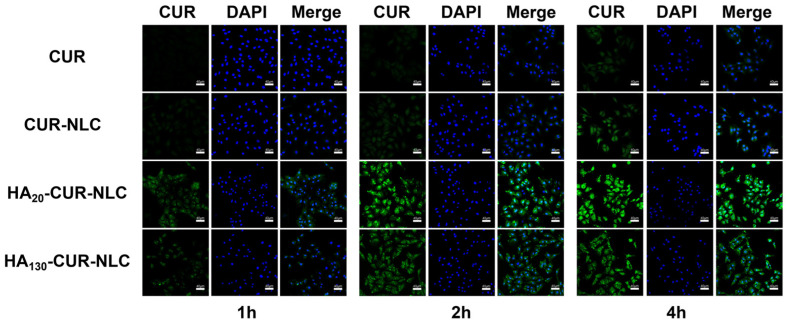
CLSM images of A549 cells after incubating with CUR, CUR-NLC, HA_20_-CUR-NLC, and HA_130_-CUR-NLC for 1, 2, and 4 h.

As presented in Figure 13, the fluorescence intensity of all groups was enhanced during the incubation time. Nevertheless, after incubation with the CUR group for 4 h, faint green fluorescence was detected inside cells, whereas that in the CUR-NLC group was markedly higher. The much stronger green fluorescence in the cytoplasm of HA_20_-CUR-NLC and HA_130_-CUR-NLC groups demonstrated that the cellular uptake effect was highly enhanced compared with unmodified NLC at each time point. This also distinctly confirmed the crucial role of HA in targeting the tumor and penetrating process; the modification of HA in KA-NLC may be responsible for efficient internalization into NSCLC via CD44 receptor-mediated endocytosis.

Meanwhile, the possible mechanism is that when HA-modified preparations were taken up into the cells, HA was broken down by hyaluronidase, then exposed the cationic core, in which ammonia groups in PEI were protonated, leading to the occurrence of the proton sponge effect and the rupture of the lysosomal membrane [42]. Finally, the drug was released into the cytoplasm or nucleus. Nonetheless, the transport mechanism of the intracellular drugs will need further investigation. However, the fluorescence intensity of the HA_130_-CUR-NLC group was markedly lower than that of the HA_20_-CUR-NLC group at the predetermined times. This result corresponds with the other assays, which confirmed the superior targeting ability of HA_20_-CUR-NLC for A549 cells.

### 2.13. The Influence of Different Preparations on EMT

Lung cancer metastasis is a complex and multistep process; invasion and metastasis processes always exist in the early stages. An integral role of epithelial-mesenchymal transition (EMT) is the significant advance of the progression of malignant tumors [43]. EMT is mainly characterized by the absence of epithelial cell phenotypic markers (E-cadherin) and the acquisition of mesenchymal cell phenotypic markers (N-cadherin and Vimentin). E-cadherin is a major molecule that mediates the tight junctions between homotypic cells. The main reason for the downregulation of E-cadherin expression is the conversion of N-cadherin by the conversion of calmodulin during EMT, which has an indispensable connection with tumor progression, metastatic potential, and poor prognosis in cancer. Meanwhile, Vimentin is an intermediate fibrous-type protein that increases the motility of the tumor cells and is highly expressed when the EMT process occurs. The expressions of EMT-related proteins were appraised by Western blot assay to preliminarily investigate the possible mechanism of preparations against A549 cells.

**Figure 14:**
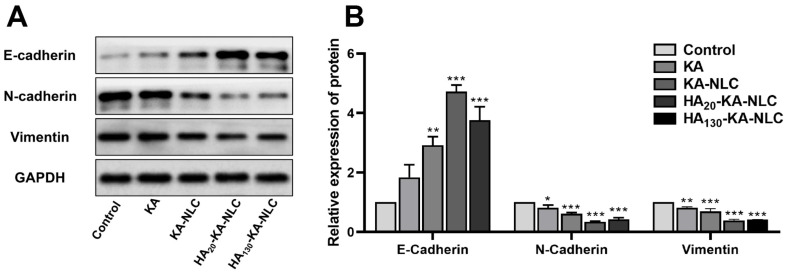
(**A**) The proteins levels of EMT-associated biomarkers untreated or treated with KA, KA-NLC, HA_20_-KA-NLC, and HA_130_-KA-NLC examined by Western blot assay. (**B**) Quantification of E-cadherin, N-cadherin, and Vimentin in A549 cells. The data are presented as the mean ± SD (*n* = 3). * *p* < 0.05, ** *p* < 0.01, *** *p* < 0.001.

As shown in Figure 14, the relative expression level of the control group was set to 100%. The relative expression of E-cadherin in KA, KA-NLC, HA_20_-KA-NLC, and HA_130_-KA-NLC groups were approximately 1.84, 2.92, 4.72, and 3.76 times higher than in the control group, respectively. Additionally, the relative expression of N-cadherin was 0.82, 0.62, 0.35, and 0.42-fold in the KA, KA-NLC, HA_20_-KA-NLC, and HA_130_-KA-NLC groups, respectively, compared with the control group. Finally, the relative expression of Vimentin in the KA, KA-NLC, HA_20_-KA-NLC, and HA_130_-KA-NLC groups was approximately 81.36%, 69.17%, 38.75% and 41.36%, respectively.

Briefly, the treatment of different preparations resulted in the upregulation of E-cadherin, while the expression of N-cadherin and Vimentin were downregulated. These results provide evidence that KA-NLC, HA_20_-KA-NLC, and HA_130_-KA-NLC had a stronger influence than free KA on impeding the EMT process in NSCLC cells. The reason may be that KA-NLC, HA_20_-KA-NLC, and HA_130_-KA-NLC increased cellular uptake of the drug compared with KA, thereby regulating EMT-related protein expression. Meanwhile, as seen from the results of the above experiments, the inhibitory effect of HA_20_-KA-NLC on EMT was better than that of HA_130_-KA-NLC in A549 cells.

## 3. Materials and Methods

### 3.1. Materials

Kaempferol, Glyceryl Monostearate (GMS), Oleic acid, Poloxamer 188, Curcumin (CUR), Poly (Ethyleneimine) (PEI, M.W. 1800 Da), and Cell Counting Kit-8 (CCK-8) were purchased from Shanghai Titan Scientific Co., Ltd. (Shanghai, China). Hyaluronic acid (HA, M.W. 200–400 kDa and 1300–1600 kDa) was obtained from Shandong Freda Biotechnology Co., Ltd. (Jinan, China). A549 cell lines were obtained from Procell Life Science & Technology Co., Ltd. (Wuhan, China). Ham’s F-12K medium was purchased from Bio-Channel Biotechnology Co., Ltd. (Nanjing, China). Fetal bovine serum (FBS) was bought from Zhejiang Tianhang Biotechnology Co., Ltd. (Hangzhou, China). EdU Cell Proliferation Kit with Alexa Fluor 594, crystal violet, Hoechst 33342, DAPI, Enhanced BCA Protein Assay Kit, RIPA lysis buffer, and Ultra hypersensitive ECL chemiluminescence kit was purchased from Beyotime Biotechnology Co., Ltd. (Shanghai, China). Anti-E-cadherin rabbit polyclonal antibody, anti-N-cadherin rabbit polyclonal antibody, anti-Vimentin rabbit polyclonal antibody, horseradish peroxidase (HRP)-conjugated affinipure goat anti-rabbit IgG (H + L) were purchased from Proteintech Group, Inc. (Wuhan, China). Anti-GAPDH rabbit polyclonal antibody was bought from Affinity Biosciences Co., Ltd. (Changzhou, China). Five SD male rats (200 ± 20 g) were obtained from the Laboratory Animals Research Center of Mudanjiang Medical University (Mudanjiang, China).

### 3.2. Preparation of KA-Loaded NLC

In this experiment, GMS with a phase transition temperature of 67 °C, and oleic acid with a phase transition temperature of 8 °C, were utilized as solid and liquid lipid, respectively. KA-NLC was prepared by the melting ultrasonic method [44] according to the following procedure; 0.02 g KA, 0.45 g GMS, and 0.15 g oleic acid were mixed and heated under magnetic stirring as the oil phase; 0.3 g Poloxamer 188 was dissolved and heated in 30 mL ultrapure water as the aqueous phase. The aqueous phase was gently and evenly introduced to the oil phase with continuous stirring to make the primary emulsion once both the oil and aqueous phases had completely dissolved and the temperature had reached 80 °C. Further, the obtained emulsion was dispersed evenly by a JY92-II ultrasonic cell grinder (Ningbo Xinzhi Biological Technology Co., Ltd., Ningbo, China) and then chilled and solidified at −20 °C to acquire KA-NLC.

The blank-NLC was prepared as described above without the addition of KA.

NLC loaded with CUR was made in the same way, but with CUR instead of KA, and utilized in the cellular uptake experiment.

### 3.3. Optimization of KA-NLC

The preparation process is one of the most vital components in the effective production of the NLC formulation. Many variables of formulation and process parameters influenced the result of EE% and DL%, such as the ratio of lipid, surfactant concentration, mixing speed, ultrasonic time, and ultrasonic power. Based on the preliminary experiments, the three main factors that affected the EE% and DL% were used to further optimize the formulation by the Box-Behnken Design (BBD) method [45]. The three independent factors (the ratio of lipid and drug (20:1–40:1)-A, the ratio of solid lipid and liquid lipid (3:1–5:1)-B, surfactant concentration (1–1.5%, *w*/*v*)-C) were observed for two dependent factors (EE%-Y_1_, and DL%-Y_2_); Table 2 shows the experimental range of the factors as well as the response constraints.

### 3.4. Characterization of NLC

#### 3.4.1. Particle Size, Polydispersity Index, and Zeta Potential Analysis

The average particle size distribution and polydispersity index (PDI) were evaluated by the dynamic light scattering method. The Zeta potential of preparations was measured by the Zetasizer Nano-ZS90 (Malvern Instruments, Malvern, UK).

#### 3.4.2. DSC Analysis

The thermal behavior of KA, GMS, the physical mixture of KA and GMS, and blank-NLC and KA-NLC were analyzed by DSC-100L differential scanning calorimetry (Nanjing Dazhan Electrical Technology Company, Nanjing, China). Each sample was accurately weighed, packed in an aluminum pan, and scanned at a temperature of up to 360 °C (rate 10 °C/min) in Nitrogen. The DSC curve was obtained using the empty aluminum pan as a reference [46].

#### 3.4.3. Transmission Electron Microscopy

To characterize the surface morphology and internal structure of preparations, a handful of preparations were diluted 10 times and added to the carbon-covered copper grid (230 mesh). The grid was dried at 37 °C for 30 min, then carried out by JEM-2100 Plus transmission electron microscopy (JEOL, Tokyo, Japan) [47].

### 3.5. Preparation of HA-KA-NLC

The modification of HA through the active electrostatic adsorption method. The cationic polymer PEI was chosen as the intermediary for connection, providing the cationic core, following absorption of the anionic HA. High molecular weight PEI (>25 kDa) has a slow systemic clearance process, which may lead to the accumulation of the polymer in the body. However, low molecular weight PEI (≤2000 Da) can be more easily eliminated from the body, which has superior degradability and less cytotoxicity compared with high molecular weight PEI [48]. So, we elected to use 1800 Da PEI for the modification of the carrier. HA-KA-NLC was prepared by adding PEI to the oil phase; the remaining steps are the same as above. Finally, HA solutions were added to the suspension under stirring. The change of Zeta potential determined the optimal concentration of PEI and HA.

### 3.6. Entrapment Efficiency and Drug Loading of Preparations

Ultrafiltration centrifugation technique [49] was used to determine the EE% and DL%. A total of 2 mL KA-NLC, HA_20_-KA-NLC, and HA_130_-KA-NLC was added into the ultrafiltration centrifuge tube and centrifuged at 10,000 rpm for 15 min; then, the ultrafiltrate was analyzed by a UV-1800 UV-Visible spectrometer (Shimadzu, Tokyo, Japan) at 268 nm to obtain the weight of free KA. Thereafter, the same batch of KA-NLC was diluted with methanol, and the total weight of KA was measured. Ultimately, EE% and DL% were computed as follows:

(1) $$\mathrm{EE}\%=\frac{\left(\mathrm{Total}\;\mathrm{weight}\;\mathrm{of}\;\mathrm{KA}\;\mathrm{added}-\mathrm{weight}\;\mathrm{of}\;\mathrm{free}\;\mathrm{KA}\right)\;}{\mathrm{Total}\;\mathrm{weight}\;\mathrm{of}\;\mathrm{KA}\;\mathrm{added}}\times 100\%$$

(2) $$\mathrm{DL}\%=\frac{\left(\mathrm{Total}\;\mathrm{weight}\;\mathrm{of}\;\mathrm{KA}\;\mathrm{added}-\mathrm{weight}\;\mathrm{of}\;\mathrm{free}\;\mathrm{KA}\right)}{\left(\mathrm{Total}\;\mathrm{weight}\;\mathrm{of}\;\mathrm{KA}\;\mathrm{added}-\mathrm{weight}\;\mathrm{of}\;\mathrm{free}\;\mathrm{KA}+\mathrm{weight}\;\mathrm{of}\;\mathrm{lipid}\right)}\times 100\%$$

### 3.7. Stability of Preparations

The optimally prepared KA-NLC, HA_20_-KA-NLC, and HA_130_-KA-NLC were stored at room temperature for 1 month for the stability study, and the relevant parameters were measured.

### 3.8. In Vitro Drug Release Study

The in vitro drug release behaviors of different preparations were investigated by the dialysis technique. A total of 3 mL of KA-NLC, HA_20_-KA-NLC, and HA_130_-KA-NLC suspensions was incorporated into the pre-treated dialysis membrane. Then, the dialysis membrane was incubated in buffer media, which was PBS with pH 5.5 and pH 7.4 [50]. The samples were tested at 37 °C with continuous stirring, and 1 mL of the buffer media was withdrawn at 1, 2, 3, 4, 5, 6, 8, 10, 14, 18, and 24 h, and an equal amount of fresh buffer media was added. Subsequently, the drug content was calculated at each time point using a UV-Visible spectrometer at 268 nm and then plotted the in vitro release curve.

### 3.9. Cell Culture

A549 cells were cultured in Ham’s F-12K medium containing 100 U/mL penicillin and streptomycin, 10% FBS at 37 °C, and 5% CO_2_ [51]. When cell confluency reached 70–80%, 0.25% trypsin was used for cell passage.

### 3.10. Safety Evaluation of Preparations

#### 3.10.1. Cytotoxicity Study

The cytotoxicity of blank-NLC was investigated using the Cell Counting Kit-8 (CCK-8) assay [52]. A549 cells were inoculated into 96-well plates (5000 cells/well) and cultured overnight at 37 °C and 5% CO_2_. Then, the blank-NLC suspension with concentrations of 0, 5, 10, 20, 40, 60, 80, 100, and 120 µmol/L was added into the wells for 24 h. There were 5 wells in each group. After removing the drug-containing medium, 100 µL of incomplete medium containing 10% CCK-8 was left to incubate for 4 h. Untreated cells were considered as control, and the medium served as the blank background. Subsequently, using a SpectraMax M3 microplate reader (Molecular Devices, San Jose, CA, USA), the OD value was obtained at 480 nm; then, the cell viability was calculated.

(3) $$\mathrm{Cell}\;\mathrm{viability}\;\left(\%\right)=\frac{{\mathrm{OD}}_{\mathrm{test}}-{\mathrm{OD}}_{\mathrm{blank}}}{{\mathrm{OD}}_{\mathrm{control}}-{\mathrm{OD}}_{\mathrm{blank}}}\times 100\%$$

#### 3.10.2. Hemolysis Activity

Red blood cell suspensions (RBCs) with blank-NLC, KA-NLC, HA_20_-KA-NLC, and HA_130_-KA-NLC were observed to evaluate the safety of hemolysis. The animal test was done according to the guidelines of the Animal Care and Ethics Committee of Mudanjiang Medical University (authorization number: 20210326-9). Fresh mouse blood was obtained from SD male rats; then, the blood was placed in an anticoagulation tube and centrifuged at 3000 rpm for 8 min until the supernatant was clear. The solid deposits were diluted with physiological saline to make 3% RBCs. An appropriate amount of physiological saline, blank-NLC, KA-NLC, HA_20_-KA-NLC, HA_130_-KA-NLC, and ultrapure water was mixed with RBCs at 37 °C for 3 h, respectively, then centrifuged for 5000 rpm for 3 min. Then, the OD value of the supernatant was measured by a SpectraMax M3 microplate reader at 540 nm, and the hemolysis percentage of samples was calculated using the following equation. The RBCs incubated with physiological saline and ultrapure water served as the negative and positive control, respectively [53].

(4) $$\mathrm{Hemolysis}\;\mathrm{percentage}\;\left(\%\right)=\frac{{\mathrm{OD}}_{\mathrm{test}}-{\mathrm{OD}}_{\mathrm{negative}\;\mathrm{control}}}{{\mathrm{OD}}_{\mathrm{positive}\;\mathrm{control}}-{\mathrm{OD}}_{\mathrm{negative}\;\mathrm{control}}}\times 100\%$$

### 3.11. Cell Viability Assay

The viability of cells treated by different preparations was investigated using the CCK-8 assay [54]. A549 cells were inoculated and cultivated into 96-well plates (5000 cells/well) for 12 h; then, 0, 5, 10, 20, 40, 60, 80, 100, and 120 µmol/L of KA, KA-NLC, HA_20_-KA-NLC, and HA_130_-KA-NLC were added. After 24 h, 100 µL of medium comprised of 10 µL CCK-8 was added for 4 h; then, the OD value at 480 nm was measured to calculate the cell viability [55].

### 3.12. EdU Staining Assay

The CCK-8 assay was used to assess the whole proliferation status of the cells, while the EdU incorporation assay can detect individual cells in a proliferative state [56], which could directly reflect the anti-proliferative effect of the preparations on A549 cells. The cells were incubated in 24-well plates (5 × 10^5^ cells/well) and cultured for 12 h at 37 °C and 5% CO_2_. After that, the cells were treated with serum-free medium containing equal concentrations of KA, KA-NLC, HA_20_-KA-NLC, and HA_130_-KA-NLC for 4 h. An EdU kit was utilized to perform the EdU assay; then, the cells were fixed with 4% paraformaldehyde for 10 min and stained for 20 min in darkness with DAPI solution. After PBS was washed, the cell proliferation was observed and photographed by a FV3000 confocal laser scanning microscope (CLSM, Olympus, Tokyo, Japan); then, the positive cell rate was calculated.

(5) $$\mathrm{Positive}\;\mathrm{cell}\;\mathrm{rate}\;\left(\%\right)=\frac{\mathrm{Positive}\;\mathrm{cells}\;\mathrm{number}}{\mathrm{Total}\;\mathrm{cells}\;\mathrm{number}}\times 100\%$$

### 3.13. Clone Formation Assay

Clone formation assay was performed to explore the inhibitory ability of different preparations in the growth of tumor cells [57]. A549 cells were inoculated into 6-well plates (500 cells/well), with the treatment of KA, KA-NLC, HA_20_-KA-NLC, and HA_130_-KA-NLC for 14 days. The formative clones were stained with 0.1% crystal violet for 5 min; then, the number of clones was observed and counted.

### 3.14. Wound Healing Assay

A549 cells were inoculated into 6-well plates (3 × 10^5^ cells/well). When the cell confluency reached about 80–90%, wound scratches with equal distance were generated by a 10 µL tip at 0 h [58]. After 12 h and 24 h post-culture of cells with KA, KA-NLC, HA_20_-KA-NLC, and HA_130_-KA-NLC, the areas of the wound scratches were calculated under a Leica DM IL LED microscope (Leica, Wetzlar, Germany).

(6) $$\mathrm{Migration}\;\mathrm{rate}\;\left(\%\right)=\frac{\mathrm{Wound}\;\mathrm{area}\;\mathrm{at}\;12\;\mathrm{or}\;24\;h}{\mathrm{Wound}\;\mathrm{area}\;\mathrm{at}\;0\;h}\times 100\%$$

### 3.15. Transwell Assay

Cell migration assay: 500 µL of serum-containing culture medium was added into the 24-well plates, and 200 µL of cell suspension with serum-free medium containing KA, KA-NLC, HA_20_-KA-NLC, and HA_130_-KA-NLC was added to the upper chamber (5 × 10^4^ cells) [59]. After incubation for 24 h, the uninvaded cells of the upper layer were wiped away, and the chamber was fixed with 4% paraformaldehyde for 15 min and stained with 0.1% crystal violet for 15 min. Finally, the stained cells in 5 visual fields were randomly counted and photographed under microscope. Cell invasion assay: diluted Matrigel covered the membrane of the chamber for 4 h prior to the procedure; the remaining steps were identical to the migration assay.

### 3.16. Apoptosis Assay

A549 cells were inoculated into 24-well plates (5 × 10^4^ cells/well); then, KA, KA-NLC, HA_20_-KA-NLC, and HA_130_-KA-NLC were added to the wells for 4 h. Then, they were fixed with 4% paraformaldehyde for 15 min, stained with Hoechst 33,342 for 20 min, and the apoptosis morphological alteration of the cell nucleus was observed by CLSM [60].

### 3.17. Cellular Uptake Assay

To explore the internalized effect of the NLC and HA modified NLC on A549 cells, the cellular uptake assay was evaluated by CLSM using CUR as a fluorescent probe. Briefly, the CUR-loaded NLC was similarly prepared, while KA was replaced with CUR. A549 cells were incubated in 24-well plates (3 × 10^5^ cells/well) and cultured for 12 h at 37 °C and 5% CO_2_. Then, CUR, CUR-NLC, HA_20_-CUR-NLC, and HA_130_-CUR-NLC of the same concentration were co-cultured with cells for 1, 2, and 4 h. Afterward, the cells were fixed with 4% paraformaldehyde for 10 min and stained with DAPI for 5 min [61]. Finally, the images of cellular uptake of different preparations were photographed by CLSM.

### 3.18. Western Blot Assay

In order to preliminarily clarify the antitumor mechanism of preparations, the protein expression levels of E-cadherin, N-cadherin, and Vimentin were evaluated by Western blot assay. A549 cells were inoculated into 6-well plates (3 × 10^5^ cells/well), with the treatment of KA, KA-NLC, HA_20_-KA-NLC, and HA_130_-KA-NLC for 24 h. The total proteins were extracted from the cells using RIPA lysis solution and determined by the BCA method, and SDS-PAGE was conducted to segregate proteins by their molecular weight. Next, the proteins were deposited onto PVDF membranes, which were blocked for 2 h with 5% defatted milk [62]. Finally, after the primary antibodies and secondary antibody incubated, the proper enhanced chemiluminescent (ECL) reagent was used to visualize the protein blot, and Image J software (National Institutes of Health, Bethesda, MD, USA) was used for quantification.

### 3.19. Statistical Analysis

All data were expressed as mean ± SD (*n* ≥ 3) and determined by one-way analysis of variance (ANOVA) [63] using GraphPad-Prism 7.0 software (GraphPad Software, La Jolla, CA, USA). *p* < 0.05 was considered statistically significant (* *p* < 0.05, ** *p* < 0.01, *** *p* < 0.001).

## 4. Conclusions

In the present study, KA-NLC was produced by the melting ultrasonic method, and the optimized nanoparticles showed suitable Zeta potential, low particle size, narrow PDI distribution, superior stability, and high DL% and EE%. Subsequently, KA-NLC was modified by HA with two molecular weights using the electrostatic adsorption method for targeting NSCLC. Then, the drug release pattern of KA-NLC, HA_20_-KA-NLC, and HA_130_-KA-NLC showed a sustained release, indicating uniform encapsulation of KA in preparations. According to the in vitro pharmacodynamic assays, HA_20_-KA-NLC and HA_130_-KA-NLC demonstrated dramatic antitumor and tumor-targeting effects on A549 cells. Furthermore, the expression of EMT-associated proteins was significantly modulated in the Western blot assay, indicating that preparations might also exert antitumor activity via suppressing EMT in A549 cells. The in vitro antitumor effect of HA_20_-KA-NLC should be noted; it was significantly stronger than that of HA_130_-KA-NLC. In summary, the selectively targeted delivery carrier we synthesized showed superiority in promoting the treatment efficiency of NSCLC, which might be a novel and promising approach for future clinical investigations of lung cancer. However, the present study didn’t involve the in vivo experiments; thus, we intend to inject NSCLC cells into mice for tumorigenic experiments and further investigate the effect of the preparations on the NSCLC of mice in future study.

## Figures and Tables

**Table 1:** Matrix and response values for the BBD method for KA-NLC optimization.

Std Order	Run Order	Factors			Responses	
		A	B	C	Y_1_	Y_2_
1	4	20	3	1.25	78.57 ± 2.22	2.90 ± 0.23
2	3	40	3	1.25	82.26 ± 2.01	2.01 ± 0.11
3	7	20	5	1.25	80.76 ± 1.58	3.24 ± 0.32
4	5	40	5	1.25	83.34 ± 2.64	2.01 ± 0.18
5	9	20	4	1	83.87 ± 1.32	4.32 ± 0.42
6	6	40	4	1	89.19 ± 1.65	2.23 ± 0.33
7	15	20	4	1.5	82.45 ± 2.62	3.77 ± 0.46
8	17	40	4	1.5	85.48 ± 2.91	2.06 ± 0.19
9	13	30	3	1	88.71 ± 3.01	2.96 ± 0.34
10	2	30	5	1	87.57 ± 2.87	2.98 ± 0.49
11	8	30	3	1.5	84.69 ± 1.94	2.89 ± 0.37
12	1	30	5	1.5	83.35 ± 1.68	2.10 ± 0.16
13	14	30	4	1.25	85.23 ± 1.54	2.86 ± 0.25
14	16	30	4	1.25	85.58 ± 2.84	2.87 ± 0.29
15	10	30	4	1.25	85.33 ± 2.46	2.61 ± 0.26
16	12	30	4	1.25	85.50 ± 2.18	2.80 ± 0.34
17	11	30	4	1.25	85.81 ± 2.73	2.82 ± 0.42

A: ratio of lipid and drug; B: ratio of solid lipid and liquid lipid; C: surfactant concentration; Y_1_ = EE (%); Y_2_ = DL (%).

**Table 2:** Factors and responses for the BBD method.

Variables	Levels Used		
	Low	Medium	High
Factors:			
A = the ratio of lipid and drug	20:1	30:1	40:1
B = the ratio of solid lipid and liquid lipid	3:1	4:1	5:1
C = surfactant concentration (%, *w*/*v*)	1	1.25	1.5
Responses:	Constraints		
Y_1_ = EE (%)	Maximize		
Y_2_ = DL (%)	Maximize		

## Data Availability

Not applicable.

## Sample Availability

Samples of the compounds are available from the authors on demand.
