# Correction: Ma et al. Hyaluronic Acid Modified Nanostructured Lipid Carrier for Targeting Delivery of Kaempferol to NSCLC: Preparation, Optimization, Characterization, and Performance Evaluation In Vitro. *Molecules* 2022, *27*, 4553

**DOI:** 10.3390/molecules29235762

**Published:** 2024-12-06

**Authors:** Yufei Ma, Jinli Liu, Xinyu Cui, Jiafu Hou, Fengbo Yu, Jinghua Wang, Xiaoxue Wang, Cong Chen, Lei Tong

**Affiliations:** 1 Department of Pharmacy, Mudanjiang Medical University, Mudanjiang 157000, China; mayufei693@126.com (Y.M.);; 2 Department of Basic Medicine, Mudanjiang Medical University, Mudanjiang 157000, China; 3 Department of Public Health, Mudanjiang Medical University, Mudanjiang 157000, China

## Errors in Figure

In the original publication [1], there were mistakes in Figures 10 and 11. The images of the HA_20_-KA-NLC and HA_130_-KA-NLC groups at 0 h in Figure 10A and the control group of the invasion experiment in Figure 11A were mistakenly shown during several rounds of figure assembly. The corrected Figures appear below. The authors state that the scientific conclusions are unaffected. This correction was approved by the Academic Editor. The original publication has also been updated.

**Figure 10:**
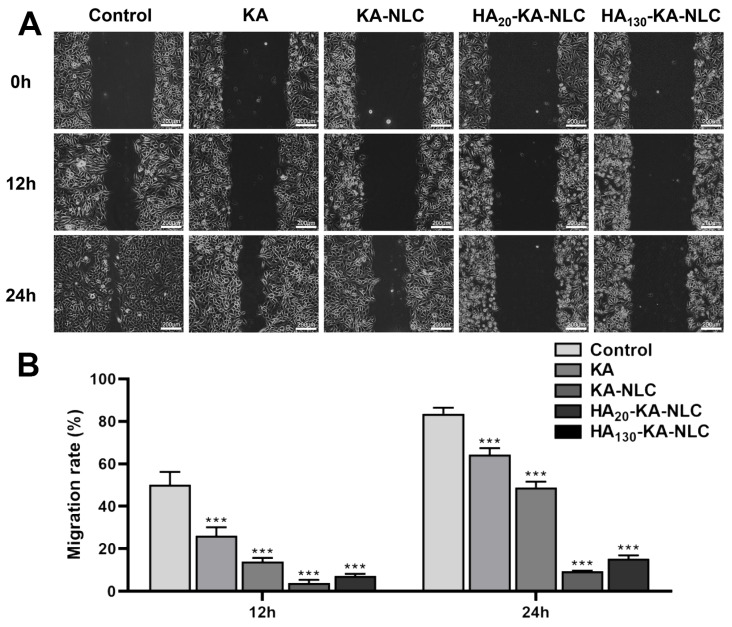
Migration assay conducted on A549 cells without treatment or with KA, KA-NLC, HA20-KA-NLC, and HA130-KA-NLC treatments. (**A**) Representative images of wound healing assays. (**B**) Migration rate of cells after different treatments. The data are presented as the mean ± SD (*n* = 3). *** *p* < 0.001.

**Figure 11:**
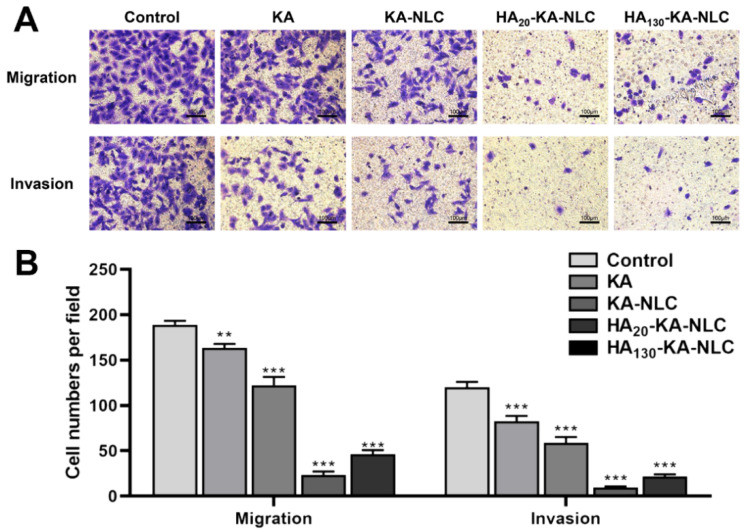
Transwell assay conducted on A549 cells without treatment or with KA, KA-NLC, HA_20_-KA-NLC, and HA_130_-KA-NLC treatments. (**A**) Representative images of migration and invasion assay. (**B**) Number of migratory or invasive cells per field after different treatments. The data are presented as the mean ± SD (*n* = 3). ** *p* < 0.01, *** *p* < 0.001.
